# The Effects of Hybrid Steel/Basalt Fibers on the Durability of Concrete Pavement against Freeze–Thaw Cycles

**DOI:** 10.3390/ma16227137

**Published:** 2023-11-12

**Authors:** Jianqiao Yu, Zijing Yi, Zhigang Zhang, Dawei Liu, Junxin Ran

**Affiliations:** 1 School of Civil Engineering, Chongqing University, Chongqing 400045, China; yujianqiao95@stu.cqu.edu.cn (J.Y.); liudawei@cqu.edu.cn (D.L.); 20184732@cqu.edu.cn (J.R.); 2 Key Laboratory of New Technology for Construction of Cities in Mountain Area, Chongqing University, Ministry of Education, Chongqing 400045, China; 3 Undergraduate School, Chongqing University, Chongqing 400045, China

**Keywords:** freeze–thaw, concrete pavements, hybrid fibers, steel fiber, basalt fiber, RDME

## Abstract

Freeze–thaw (F-T) is one of the principal perils afflicting concrete pavements. A remedial strategy used during construction encompasses the integration of hybrid fibers into the concrete matrix. An extant research gap persists in elucidating the damage mechanism inherent in hybrid steel fiber (SF)- and basalt fiber (BF)-reinforced concrete subjected to F-T conditions. This paper empirically investigated the durability performance of hybrid fiber-reinforced concrete (HFRC) subjected to F-T cycles. The impact of SF/BF hybridization on mass loss, abrasion resistance, compressive strength, flexural strength, damaged layer thickness, and the relative dynamic modulus of elasticity (RDME) was examined. The damage mechanism was explored using micro-hardness and SEM analysis. The results indicate that incorporating hybrid SF/BF effectively enhances the F-T resistance of concrete and prolongs the service life of concrete pavement. The mechanisms underlying these trends can be traced back to robust bonding at the fiber/matrix interface. Randomly dispersed SFs and BFs contribute to forming a three-dimensional spatial structure within the concrete matrix, suppressing the expansion of internal cracks caused by accumulated hydrostatic pressure during the F-T cycle. This research outcome establishes a theoretical foundation for the application of HFRC to concrete pavements in cold regions.

## 1. Introduction

In the frigid region, diurnal temperature variations engender deleterious effects on rigid materials such as concrete. These deleterious consequences are particularly manifest in the context of concrete road pavements, where they manifest as premature cracking, diminished wear resistance, and a concomitant reduction in load-bearing capacity [1,2,3]. Hence, these road pavements exhibit truncated operational lifespans and commensurately elevated maintenance expenditures. Freeze–thaw (F-T) cycles stand as a principal factor that diminishes the longevity of concrete pavements, precipitated by temperature variations [4]. As water transitions into ice, it undergoes a volumetric expansion of up to 9% of its total volume, giving rise to micro-cracks [5]. During the thawing process, water infiltrates the pavement via micro-cracks. Furthermore, during the freezing phase, water that permeates the unyielding pavement solidifies, resulting in a diminution in concrete strength due to the concomitant emergence of macro-cracks [6]. Therefore, it becomes imperative to ascertain means to mitigate the propensity for concrete cracking to enhance the endurance of road pavement against the challenges of F-T cycles.

The incorporation of fibers into concrete can effectively mitigate, regulate, and retard the onset, progression, and merging of minute and substantial cracks [7,8,9], thereby addressing several drawbacks associated with concrete [10,11,12] and minimizing impairment in F-T conditions [13,14]. The contribution of fibers to concrete exhibits variability based on their shape, length, density, elastic modulus, and tensile strength. Longer fibers exert a profound influence on the inception, advancement, and amalgamation of major cracks [15], whereas shorter fibers distinctly determine micro-scale cracks [16,17]. Accordingly, the hybridization of two distinct fiber types likely optimizes their respective merits [18]. Consequently, in recent years, there has been a burgeoning interest in the application of hybrid fiber-reinforced concrete (HFRC) within road pavement.

Among fiber hybridizations, the combination of steel fiber (SF) and basalt fiber (BF) stands as an innovative prospect with vast applicability [19]. Guler et al. [20] conducted an inquiry into the properties of concrete pavement with the incorporation of hybrid fibers within the concrete matrix. The concrete specimens featuring SFs and BFs exhibited superior flexural strength and impermeability properties in comparison with ordinary concrete. The outcomes revealed that the propagation of cracks within the concrete pavement was notably restrained with the integration of hybrid fibers. Zhang et al. [21] investigated the dynamic properties of strain-hardening cementitious composites (SHCCs) incorporating BF and SF. It was observed that the SHCC incorporated with hybrid fibers evinced a pronounced superiority concerning its initial crack, post-cracking response, and heightened potential for energy absorption when compared with conventional plain concrete. Khan et al. [22] demonstrated that the hybridization of SFs and BFs serves to augment the mechanical properties of HFRC. Particularly, the optimal volumetric blend of 0.32% SF and 0.68% BF resulted in a maximal enhancement in compressive and flexural strength in the concrete. This substantiates the efficacy of co-blending steel fibers and basalt fibers in fortifying the mechanical performance of concrete, capitalizing on the commendable elastic modulus of SFs and the noteworthy tensile strength of BFs [23]. Furthermore, these fibers have been proven advantageous in bolstering concrete durability [24,25] and reducing costs [26]. Meanwhile, it has been observed that the durability deterioration of concrete during F-T cycles is a multifaceted and multiscale process, characterized by the substantial accrual of crack propagation [27,28]. Hence, it is eminently judicious to use a combination of SFs and BFs to leverage their synergistic effect across different structural factors and stress phases of concrete and improve the durability against the F-T of concrete.

In light of the antecedent literature survey, there exists a dearth of investigations dedicated to the comprehensive evaluation of the durability of hybrid SF/BF-reinforced concrete subsequent to exposure to F-T cycles, especially when juxtaposed with conventional concrete for utilization in rigid pavement applications. Furthermore, the explication of the damage mechanism and the laws governing damage evolution have not been sufficiently elucidated, leading to an insufficient comprehension of the durability and predictive lifespan of HFRC within F-T regions.

This study aims to develop a composite fiber-reinforced concrete made of hybrid SF/BF. The intention is to deploy this composite in road engineering projects in frigid climates, endowing it with superior resistance to F-T cycles compared with conventional concrete road surfaces. Specifically, the F-T deterioration patterns in the HFRC were analyzed comprehensively, encompassing both macroscopic and microscopic dimensions, while also engaging in an in-depth exploration of the damage mechanisms inherent to HFRC during the F-T process. Finally, based on the test results, a Grey–Markov model is used to establish a law governing the evolution of damage in HFRC, ultimately facilitating the characterization of its damage degree and prediction of the service life of HFRC materials within the context of F-T cycle environments. The findings of this study hold promise in offering valuable insights into the selection of concrete pavement materials in cold regions, concurrently enriching the variety of ingredients in HFRC.

## 2. Experimental Procedure

### 2.1. Raw Materials

**Figure 1:**
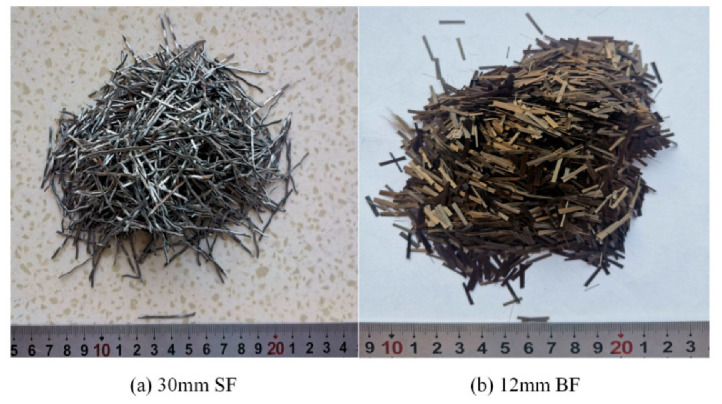
The appearances of SFs and BFs.

In this study, CEM 42.5 Portland cement, possessing a 28-day compressive strength of 49.8 MPa, was used alongside Grade I fly ash, characterized by an activity index surpassing 95%. The corresponding chemical compositions are shown in Table 1. For the concrete matrix, coarse aggregates with a needle-like flake composition and crushing indices of 7% and 9.3%, respectively, were chosen, exhibiting a granular size ranging from 5 to 20 mm. The fine aggregates featured a minimal mud content of less than 1.5% and a fineness modulus of 2.58. Two distinct types of fiber, namely, steel fiber (SF) and basalt fiber (BF), were selected for incorporation into the concrete mixture. The appearances of SFs and BFs are presented in Figure 1, while their technical attributes are delineated in Table 2. The influence of fiber on the workability of concrete mixing was enhanced with the application of a polycarboxylic acid-based superplasticizer.

### 2.2. Mix Proportion and Experimental Design

This experiment ascertained the mixture proportions using the JTG D40-2011 standard [29], as presented in Table 3. The experimental variables encompassed diverse fiber types: Group A served as the control group without fiber, Group B comprised the concrete mixture with SFs, Group C comprised the concrete mixture with BFs, and Group D incorporated hybrid SF/BF into the concrete mixture. The comprehensive details of the blending and casting procedures for the specimens were meticulously documented in a previous study [30]. The pertinent test projects and specimen dimension requirements are shown in Table 4.

**Figure 2:**
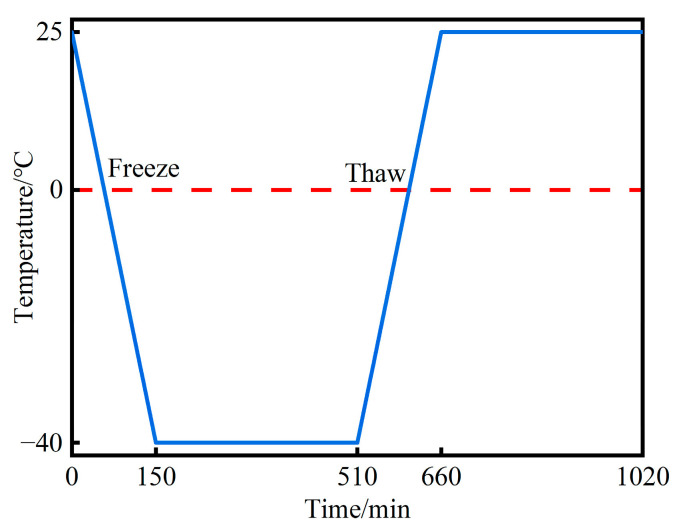
The design scheme of the F-T cycle test.

The internal time–temperature curve of concrete during F-T cycles is depicted in Figure 2. The simulated F-T damage was administered to concrete specimens that had been cured for 28 days. The specimens were first subjected to a 6-h freezing period at −40 °C, followed by a 2.5-h thawing period at 25 °C, constituting a single cycle. The corresponding tests were conducted at intervals of 25 cycles, totaling 200 cycles.

### 2.3. Test Procedures

#### 2.3.1. Mass Loss Tests

The mass loss of the specimen was determined using a precision electronic balance with a precision of 0.1 g. Measurements were conducted at regular intervals of 25 F-T cycles. The mass loss rate was utilized to characterize the specimen’s mass loss; the calculation formula is as follows:

(1) $$\Delta W_{n}=\left(1-\frac{W_{n}}{W_{0}}\right)\times 100\%$$

where ∆*W_n_* is the mass loss rate of the specimen that underwent F-T cycles, *W_n_* is the mass of the specimen after F-T cycles (g), and *W*_0_ is the initial mass of the specimen (g).

#### 2.3.2. Abrasion Resistance Tests

According to JTG 3420-2020 [31], the prepared specimen was subjected to an oven-drying regimen at 60 °C for 12 h before the abrasion resistance test. This preconditioning ensured the specimen’s optimal dry state, facilitating the subsequent testing procedure conducted under a substantial 200 N abrasion load. The assessment of the specimen’s abrasion resistance was ascertained with the utilization of the unit area abrasion loss, and the calculation formula is presented as follows:

(2) $$G_{n}=\frac{M_{0}-M_{n}}{A}$$

where *G_n_* is the abrasion loss of the specimen that underwent F-T cycles, *M*_0_ is the mass of the specimen before abrasion (g), *M_n_* is the mass of the specimen after abrasion (g), and *A* is the specimen abrasion area (m^2^).

#### 2.3.3. Compressive Strength Tests

The compressive strength of the concrete specimens was determined using a hydraulic testing machine. A load application rate of 0.5 MPa/s was steadily and continuously applied until the specimens were destroyed. The mechanical assessment index was acquired with the utilization of the compressive strength loss ratio, as per Equation (3), to facilitate subsequent calculations.

(3) $$R_{c}=\frac{R_{c}{}_{0}-R_{c}{}_{n}}{R_{c}{}_{0}}\times 100\%$$

where *R_c_* is the compressive strength loss ratio of the specimen that underwent F-T cycles, *R_c_*_0_ is the initial compressive strength of the specimen (MPa), and *R_cn_* is the compressive strength of the specimen after F-T cycles (MPa).

#### 2.3.4. Flexural Strength Tests

**Figure 3:**
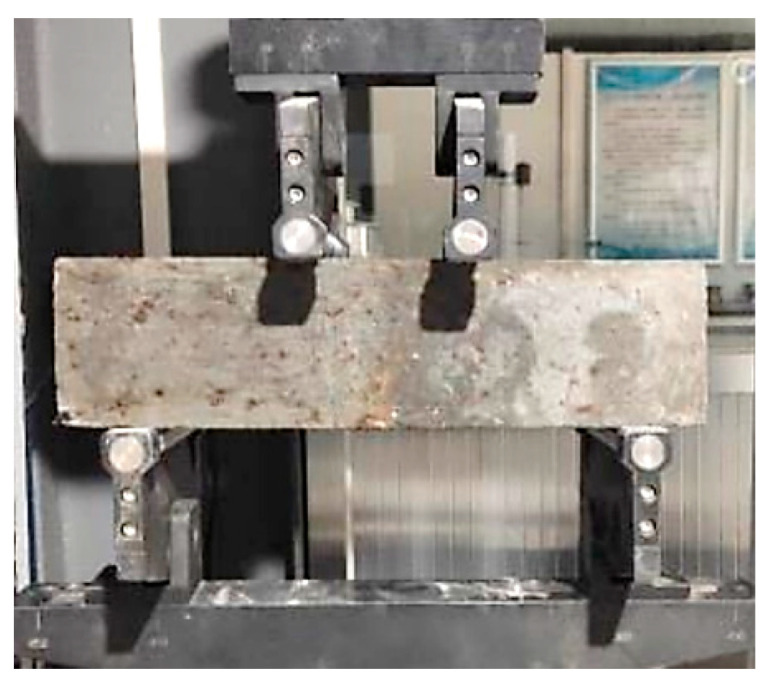
The four-point flexural test setup.

The flexural performance of concrete was scrutinized through a four-point bending test, as illustrated in Figure 3. The support spacing was set at 300 mm, with a pure bending section interval of 100 mm. During testing, displacement loading was applied at a rate of 0.002 m/s until the specimen’s failure. The primary mechanical performance indicators of pavement concrete were derived using the flexural strength loss ratio, as delineated in Equation (4).

(4) $$R_{f}=\frac{R_{f}{}_{0}-R_{f}{}_{n}}{R_{f}{}_{0}}\times 100\%$$

where *R_f_* is the flexural strength loss ratio of the specimen that underwent F-T cycles, *R_f_*_0_ is the initial flexural strength of the specimen (MPa), and *R_fn_* is the flexural strength of the specimen after F-T cycles (MPa).

#### 2.3.5. Thickness of the Damaged Layer and Relative Dynamic Modulus of Elasticity Tests

**Figure 4:**
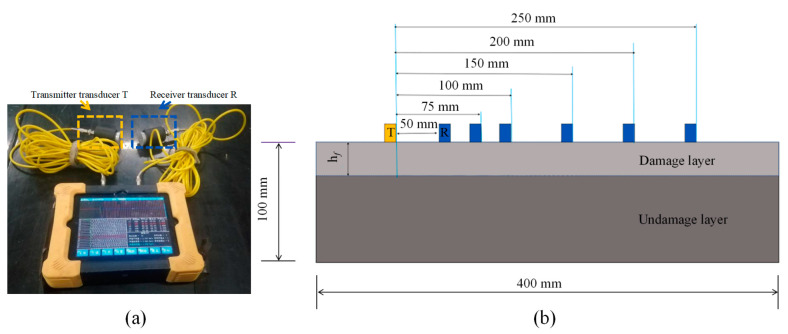
(**a**) Ultrasonic detector and (**b**) Schematic diagram of damage thickness test.

Following the completion of each F-T cycle, an ultrasonic testing device (Figure 4a) was used to assess the relatively dry regions on the concrete surface, from which the thickness of the damaged layer was ascertained. The layout of measurement points is illustrated in Figure 4b. The ultrasonic wave velocity can effectively portray the defects that emerge during the degradation process of the specimens. Previous investigations [32] have demonstrated the correlation between the relative dynamic modulus of elasticity (RDME) and ultrasonic velocity, as depicted by Equation (5).

(5) $$P_{n}={\left(\frac{V_{n}}{V_{0}}\right)}^{2}\times 100\%$$

where *P_n_* is the RDME value of the specimen subject to *n* times cyclic F-T, *V_n_* is the UPV value after *n* times cyclic F-T (m/s), and *V*_0_ is the UPV value without any F-T cycle (m/s).

#### 2.3.6. Micro-Hardness Tests

**Figure 5:**
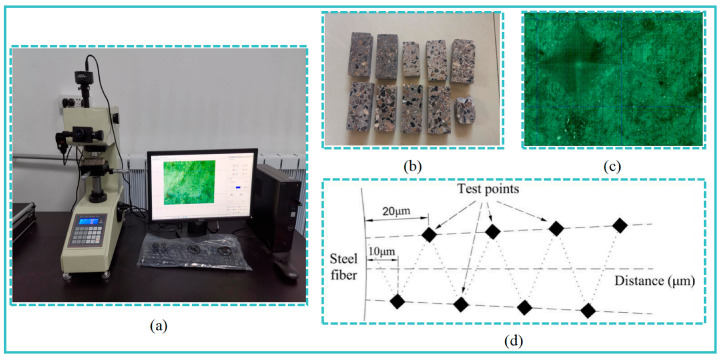
Illustration of the micro-hardness test: (**a**) Micro-hardnedd tester, (**b**) Micro-hardness test sample, (**c**) Typical shape of indentation and (**d**) Selection scheme of test points.

Based on a micro-hardness tester (Figure 5a), the measurement of micro-hardness spanning from the fiber’s edge to the bulk paste matrix was performed. Test specimens, measuring 100 mm × 40 mm × 20 mm (as shown in Figure 5b), were served as the subjects for these micro-hardness tests. The indentation patterns observed under an optical microscope are exemplified in Figure 5c. The test points were divided into two distinct parts. Within each part, a spacing of 20 μm was established between adjacent test points to eliminate overlapping. This implies that the effective step size between test points was set at 10 μm. A schematic representation of the test locations can be seen in Figure 5d. Building upon previous research [30], a force of 10 gf (0.098 N) was applied, with a dwell time of 10 s, for the micro-hardness tests.

#### 2.3.7. SEM Tests

A scanning electron microscope (SEM) was used to conduct a microscopic analysis of the concrete specimens. Tiny particle samples from the crushed specimens, both before and after F-T cycles, were extracted. A gold sprayer was used to apply a fine gold coating to the sample surfaces to enhance image quality. Subsequently, these samples were placed onto a specimen holder, ensuring electrical connectivity with the holder with the use of conductive tape. Once the sample surfaces dried, the specimens were introduced into the SEM for examination.

## 3. Results and Discussion

### 3.1. Macroscopic Results

#### 3.1.1. Surface Deterioration and Mass Loss

**Figure 6:**
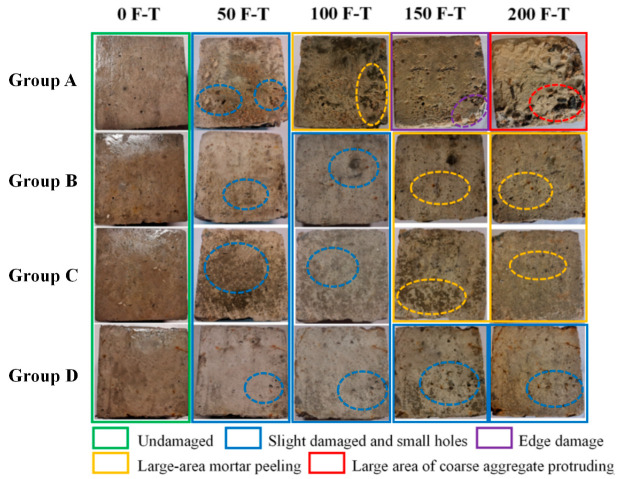
Surface deterioration of concrete samples exposed to F-T cycles.

The apparent morphological transformations of the concrete specimens under varying F-T cycles are depicted in Figure 6. It can be observed that exposure to F-T cycles destroyed the surface layers of specimens and the damage on the specimens’ surfaces became more visible with exposure to increasing F-T cycles. After 200 F-T cycles, apparently loose particles were observed on the surface of Group A, accompanied by a pronounced impairment in the edges and corners. Conversely, Groups B, C, and D exhibited noticeably milder surface erosion compared with Group A. Following 200 F-T cycles, Group B showed only a small number of minor pores on the surface, while the edge loss in Group C was comparable to that of Group B. The incorporation of hybrid SF/BF enhanced the concrete’s resistance to F-T cycles. After 200 F-T cycles, Group D displayed a favorable surface appearance, devoid of severe corner deterioration.

**Figure 7:**
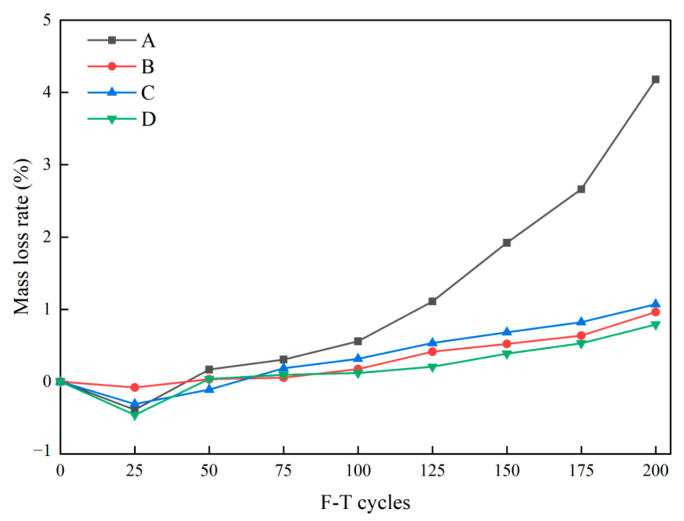
The mass loss rate of the specimens during F-T cycles.

In relation to the extent of surface damage to the concrete, the mass loss rate serves as a quantitative analytical method. The mass loss rate outcomes for each group are presented in Figure 7. As shown in Figure 7, the mass loss rate of each group exhibits an upward trend after 200 F-T cycles, except for the slight decrease at the initial 25 cycles, in which the mass of each group increases by 0.08~0.46%. This phenomenon arises from the synergistic influence of frost-induced expansion pressure and hydrostatic pressure within the specimens [33], resulting in a significant amount of water encapsulated within the pores. After 25 F-T cycles, the rate of mass loss for each specimen exhibits a gradual increase. This phenomenon ensues as the ice crystals commence their expansion, thereby precipitating the generation of external forces, which subsequently serve to amplify the pre-existing microcracks within the concrete matrix [34]. The mass loss rate of Group A upon completion of 200 F-T cycles is measured at 4.18%, whereas the other three groups demonstrate values spanning from 0.78% to 1.07%. Notably, Group D exhibits the lowest mass loss rate (0.78%). This outcome is primarily attributed to the crack-bridging capacity of the fibers and their distribution within the concrete matrix. On one hand, owing to the superior elastic modulus and sheared wavy shape, SFs have a superior bond to the surrounding matrix, thereby exhibiting enhanced crack-restrain capabilities. On the other hand, the substantial quantity of BFs dispersed randomly in the concrete matrix forms a three-dimensional confinement structure, engendering the unity of concrete. Thus, the combination of SFs and BFs distinctly contributes to preventing specimen shedding under F-T cycles.

#### 3.1.2. Abrasion Resistance

**Figure 8:**
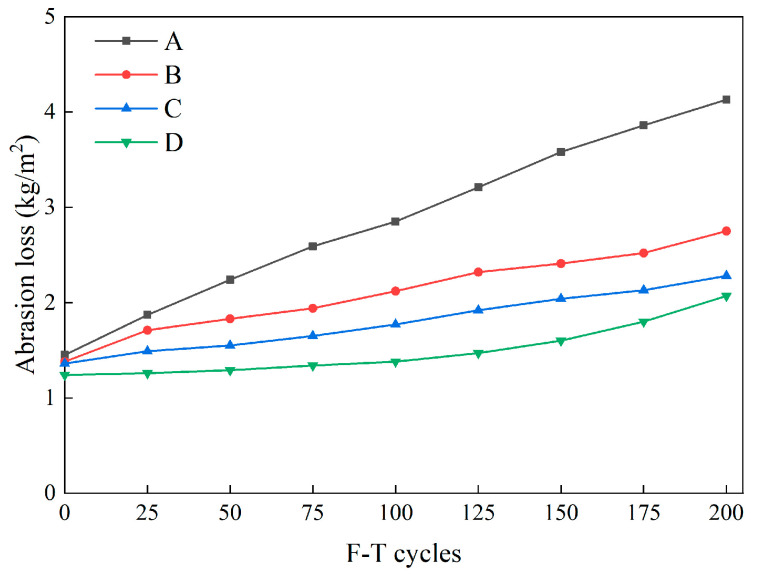
The abrasion loss of specimens during F-T cycles.

The abrasion loss of each group of concrete specimens measured after each F-T cycle is presented in Figure 8. As displayed in Figure 8, the initial abrasion loss values among the various groups exhibited minimal differences, ranging from 1.24 kg/m^2^ to 1.45 kg/m^2^. As the F-T cycles progressed, Group A witnessed a sharp increase in abrasion loss, whereas the abrasion loss rate for the three fiber-reinforced groups notably remained lower than that of the control group. Finally, after 200 F-T cycles, Group A exhibited an abrasion loss of 4.13 kg/m^2^ under a 200 N load. In contrast, the wear values for Groups B, C, and D ranged from 2.07 kg/m^2^ to 2.75 kg/m^2^, significantly lower than Group A. Notably, Group D displayed the most substantial divergence in abrasion loss when compared to the control group, with a difference of 2.06 kg/m^2^. This observation underscores that, when abrasion resistance serves as a reference criterion, HFRC exhibits at least twice the F-T resistance of conventional concrete.

Furthermore, as discerned from Figure 8, Group B, which solely incorporated SFs, exhibited slightly higher abrasion loss values throughout the entire F-T cycle period compared with Groups C and D. This phenomenon can be attributed to the fact that SFs do not effectively inhibit the formation of internal microcracks and their transformation into macroscopic cracks [35]. Consequently, with the assistance of BFs, concrete with fewer internal structural cracks better preserves its integrity and minimizes wear and tear caused by friction. Hence, Group D, which incorporated hybrid SF/BF, demonstrated outstanding abrasion resistance throughout the F-T cycles.

#### 3.1.3. Compressive Strength Loss

**Figure 9:**
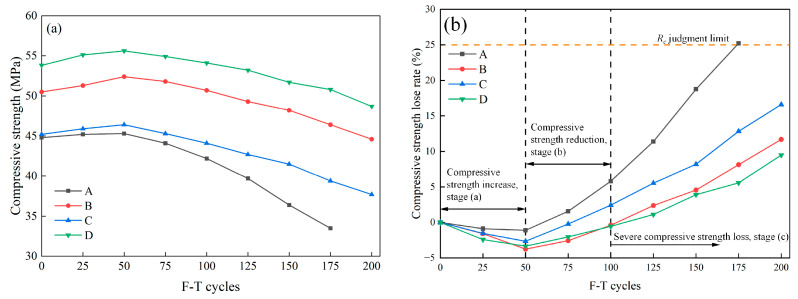
The compressive strength results of specimens after different F-T cycles: (**a**) compressive strength and (**b**) compressive strength loss rate.

The compressive strength of specimens as a function of F-T cycles is displayed in Figure 9a. In the initial phase, the compressive strength of Group A at 28 days reaches 44.8 MPa, meeting the requirements for road traffic [29]. In comparison, due to the ability of fibers to withstand additional loads when the matrix fails, the compressive strength of specimens in Groups B, C, and D, which are admixed with fibers, surpasses that of Group A, ranging between 45.2 MPa and 53.8 MPa. As the F-T cycles progress, all specimens exhibit a trend of initial increase followed by a subsequent decrease. Notably, Group D demonstrates the highest compressive strength (55.6 MPa) after 50 F-T cycles, surpassing Group A by 22.74%. After 175 F-T cycles, Group A exhibits the lowest compressive strength (33.5 MPa), indicating severe F-T damage to ordinary concrete in this experiment. Following 200 F-T cycles, the compressive strength of Groups B and D, which contain SF, is 44.6 MPa and 48.7 MPa, respectively, which is a high value for pavement concrete.

Further analyzing the compressive strength durability of HFRC, the correlation between the rate of compressive strength loss and the number of F-T cycles can be discerned (Figure 9b). As depicted in Figure 9b, following 200 F-T cycles, concrete specimens from each group exhibited a marginal increase in compressive strength compared with the initial measurements, but overall, a loss in strength was evident. The variations in compressive strength for each specimen can be categorized into three distinct stages: (a) compressive strength increase stage, (b) compressive strength reduction stage, and (c) severe compressive strength deterioration stage.

During stage (a), when the number of F-T cycles was fewer than 50, the compressive strength of each group concrete increased by approximately 1.12% to 3.76%. This elucidates that, during the early stages of F-T cycles, cement particles in the concrete were still capable of initiating hydration reactions, thereby leading to an elevation in the compactness of the cementitious matrix and an augmentation in compressive strength. In stage (b), under the expansion pressure upon F-T exposure, internal defects within each group of specimens gradually enlarged, leading to a decrease in the compactness of concrete, and consequently, reducing compressive strength. In stage (c), the rate of compressive strength loss for all groups was significantly higher than in the previous two stages. Group A exhibited an earlier failure compared with the other three groups with fiber incorporated, reaching the specified failure threshold after 175 F-T cycles. Additionally, after 200 cycles, the compressive strength loss rates for the fiber-incorporated Groups B, C, and D ranged from 9.48% to 16.59%. Among them, Group D exhibited the lowest compressive strength loss rate.

#### 3.1.4. Flexural Strength Loss

**Figure 10:**
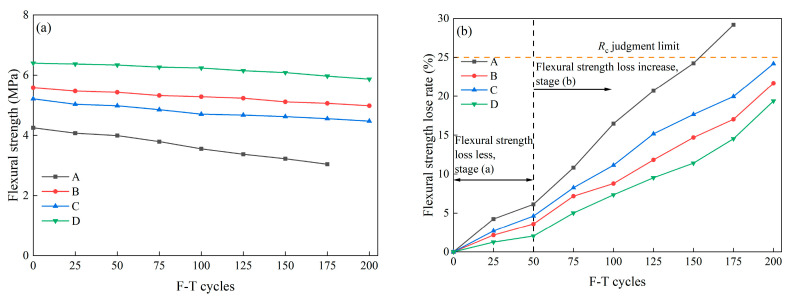
The flexural strength results of specimens after different F-T cycles: (**a**) flexural strength and (**b**) flexural strength loss rate.

Apart from compressive strength, the flexural strength of concrete is typically designated as a crucial mechanical parameter for pavement design. The curve depicting the flexural strength of concrete in relation to the number of F-T cycles obtained from a four-point bending test is illustrated in Figure 10a. In the initial phase, the flexural strength of Group A at 28 days reaches 4.25 MPa, meeting the minimum design standard (4 MPa) specified in the standard of concrete pavement maintenance technology in China [29]. The incorporation of fibers conspicuously enhances the flexural strength of concrete. The 28-day flexural strength of specimens in Groups B, C, and D ranges from 5.21 MPa to 6.40 MPa, surpassing the standard flexural strength value for cement concrete pavement under heavy loads (5 MPa). As F-T cycles progress, the flexural strength of all specimens gradually declines. In the case of Group A, the flexural strength at 175 F-T cycles is 3.01 MPa, failing to meet the requirements for highway traffic. The extent of flexural strength damage in specimens from Groups B, C, and D is markedly lower than that of ordinary concrete. Notably, the flexural strength of Group D specimens after 200 F-T cycles is 5.16 MPa, retaining an excellent load-bearing capacity.

The flexural strength loss rates of specimens from Group A to Group D are depicted in Figure 10b. As delineated in Figure 10b, the flexural strength loss rates of specimens in each group increased with the augmentation of F-T cycles, among which Group A exhibited the most pronounced deterioration. The degradation of flexural strength can be roughly categorized into two stages: (a) relatively modest flexural strength loss and (b) increases in the loss rate of flexural strength

In stage (a), the increase in flexural strength loss rate is gradual. Group A’s Rf was increased by 6.11% after 50 F-T cycles, while the magnitude of flexural strength loss for specimens from Group B to Group D ranged between 2.03% and 4.61%. In stage (b), the flexural strength loss rate for Group A specimens experienced a rapid escalation, surpassing the failure threshold of 25% at 175 F-T cycles (29.17%). In contrast, although the flexural strength loss rates from Group B to Group D exhibited an increase compared with that of stage (a), the extent of damage remained lower than that of Group A. After 200 F-T cycles, the *R_f_* values for Groups B to D ranged between 19.38% and 24.18%. Notably, Group D displayed the lowest flexural strength loss rate, indicating that the interwoven SF/BF within the concrete forms a “chaotic supportive system,” effectively impeding crack propagation induced by F-T damage and enhancing the concrete’s toughness [24].

#### 3.1.5. Damaged Layer Thickness

**Figure 11:**
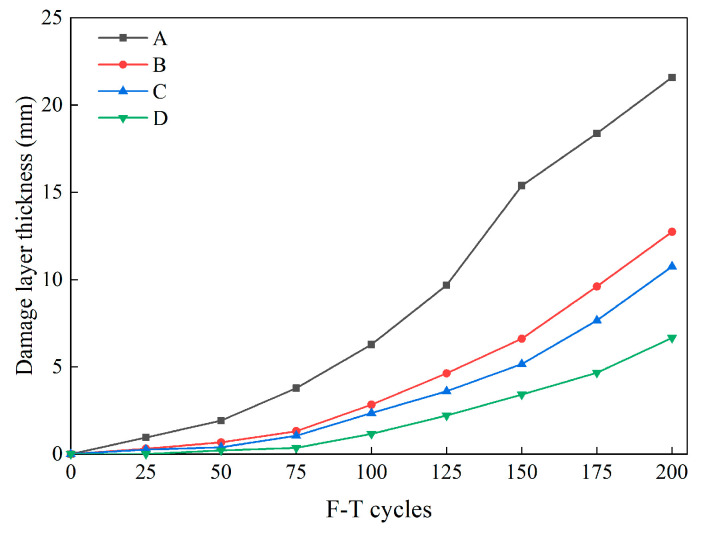
The thickness of the damaged layer of the specimens during F-T cycles.

Figure 11 illustrates the evolution of concrete damage layer thickness under the influence of F-T cycles. From the results, it is evident that F-T cycles induced varying rates of damage to the test piece. At the end of the F-T testing in Group A, the damage layer reached a substantial length of 21.59 mm, whereas the maximum thickness observed in specimens with fiber incorporation was only 12.74 mm (Group B). Under an equal number of cycles, Group B exhibited a greater thickness of the damaged layer compared with Groups C and D. After 200 F-T cycles, the damaged layer thickness for Groups C and D measured 10.75 mm and 8.93 mm, respectively. Furthermore, in conjunction with the findings from Section 3.1.1, it is discernible that if the damaged layer thickness exceeds 20 mm, it signifies concrete damage that can be visually observed with the naked eye.

Based on the aforementioned observations, it becomes evident that repeated F-T cycles expedite the degradation of the internal structure of concrete. Therefore, the incorporation of hybrid SF/BF into concrete serves to enhance its internal structural integrity, making it a promising solution for widespread use in cold-region concrete pavement applications.

#### 3.1.6. Relative Dynamic Modulus of Elasticity

**Figure 12:**
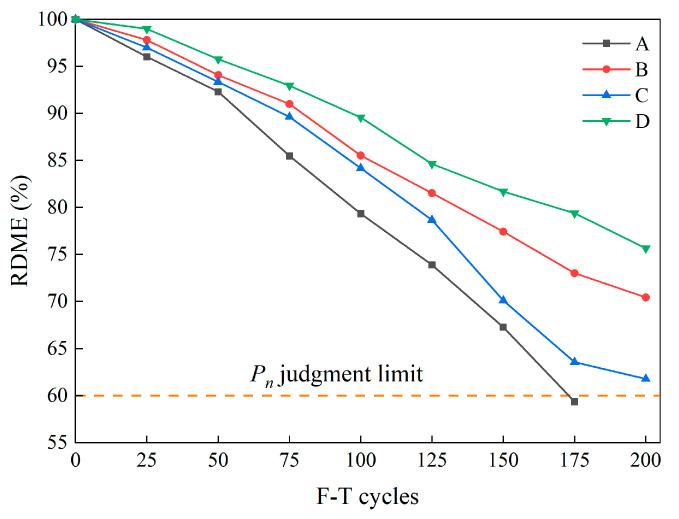
The RDME of the specimens during F-T cycles.

Figure 12 shows the change in the RDME of specimens as F-T cycles increase. It can be found that the RDME decreased significantly for all specimens after F-T cycles. Notably, the RDME of Group A exhibited a rapid decline, culminating in the attainment of the durability failure threshold after enduring 175 F-T cycles. In contrast, the RDME values for the three groups of specimens containing fibers remained above 60% after 200 F-T cycles, within the range of 61.77% to 75.65%. Specifically, before reaching 100 F-T cycles, the decline in RDME of Group C was almost the same as that of Group B. At 200 F-T cycles, the RDME of Group C was 61.77%, which was significantly lower in comparison with Group B and Group D. This indicates that in the later stages of F-T cycles, BFs exhibit a limited inhibitory effect on the macro-crack propagation caused by F-T damage. Moreover, compared with the other groups, the RDME of Group D consistently yielded the highest value, reaching 75.65% after 200 F-T cycles. This observation underscores the enhanced reinforcement of the fiber/matrix interface in concretes where SFs and BFs are hybridized, ultimately leading to better preservation of the concrete matrix integrity following F-T cycles.

### 3.2. Microscopic Results

#### 3.2.1. Micro-Hardness Test Results

**Figure 13:**
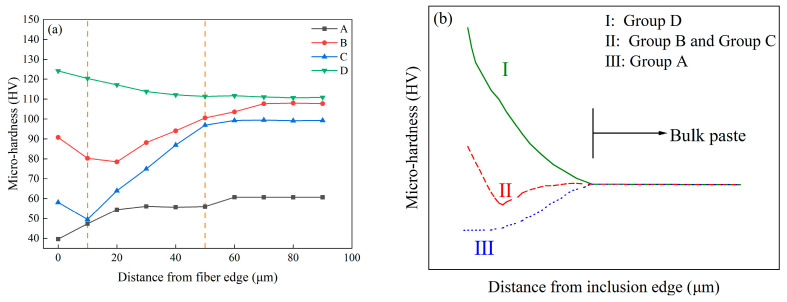
Micro-hardness values in the ITZ and typical micro-hardness trend curves of the fiber edge–matrix interfacial zone in concrete specimens: (**a**) micro-hardness around fibers and (**b**) typical micro-hardness trend curves.

Figure 13a shows the results of the micro-hardness test within 90 μm of the fiber edge for specimens with various hybrid fibers after 200 F-T cycles. The horizontal axis shows the distance from the fiber edge, whereas the vertical axis shows the micro-hardness values. Zhang et al. [36] reported that fractures induced by F-T are typically readily detected within the vulnerable interface, situated approximately 10~50 μm from the fiber interface. In this study, the division of the interfacial transition zone (ITZ) adheres to this principle.

From Figure 13a, it can be seen that, excluding Group D, the micro-hardness of the ITZ adjacent to the fiber edge in the samples exhibits inferiority to that of bulk paste located farther away from the fiber edge. This discrepancy arises due to the influence of F-T cycles, which render the micro-structure of the ITZ more porous and heterogeneous compared with the bulk paste, consequently leading to diminished bonding between fibers and the matrix within the ITZ [37]. Notably, the ITZ in Group A proves to be the feeblest, registering a mere 56.03 HV at a distance of 50 μm. However, with the progressive inclusion of SFs, the micro-hardness of the ITZ experiences an elevation. At 50 μm, the micro-hardness of Group B was increased by 79.34% compared with that of the control group at the same distance. This observation elucidates the beneficial role played by SFs in enhancing the properties of ITZ. Moreover, a denser ITZ between fibers and the matrix becomes apparent in Group D, which improves the F-T resistance of concrete [38].

Figure 13b shows three types of micro-hardness distributions around rigid inclusions in the cement matrix [39]. This categorization elucidates the relative strengths and weaknesses of the matrix near the inclusion as well as those of the matrix situated further away, thereby characterizing the toughness of the inclusion–matrix interface bond. The micro-hardness profiles of Group A exhibited Type III, indicating an absence of fiber–matrix bonding and the ITZ displaying weaker attributes. Conversely, the micro-hardness profiles of Group B and Group C exemplified Type II, suggesting a partial bond at the fiber–matrix interface. This phenomenon emerged due to the abundance of interstices and interlinked porosity proximate to the fibers, rendering the establishment of a robust bond at the interface arduous. Notably, the micro-hardness profiles of Group D demonstrated Type I, denoting an excellent bonding between the fibers and encompassing matrix. Consequently, the matrix surrounding the fibers showcased superior properties. The incorporation of SF/BF hybrid fibers resulted in a diminished disparity between the matrix neighboring fibers, and it is situated farther away.

Therefore, the micro-hardness value measured in this study for HFRC significantly surpasses the other specimens, thereby implying the superior bonding achieved with the incorporation of hybrid fibers and the consequential enhancement in F-T durability, as evidenced by mass loss, compressive strength, and RDME.

#### 3.2.2. SEM Observations

**Figure 14:**
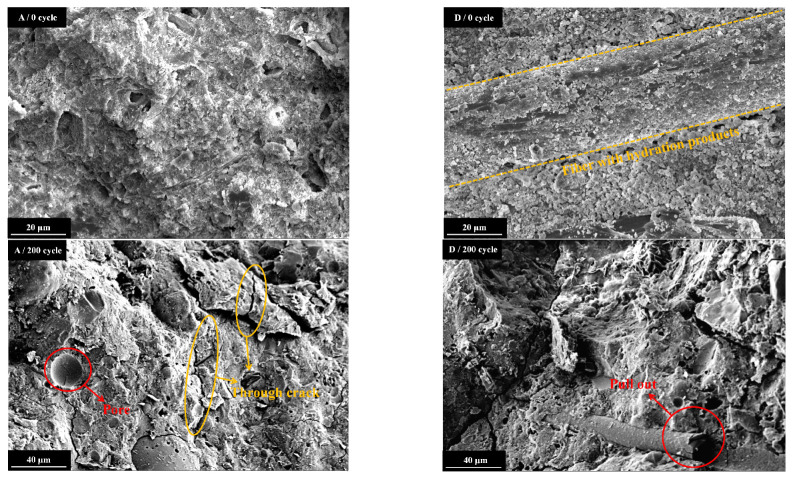
The SEM images of Group A and Group D specimens under different F-T cycles.

In this study, the micro-structure images of Group A and Group D specimens, after 0 and 200 F-T cycles, were assessed with SEM analysis. The interior structure images obtained using the SEM analysis of Group A and Group D with hybrid SF/BF-reinforced specimens are shown in Figure 14. As illustrated in Figure 14, antecedent to the inception of the F-T cycle experiment, the microstructure of Group A remained stable and undamaged. The fiber surfaces in Group D were ensconced with copious hydration products, thereby endowing the fibers with excellent bonding properties to the cementitious matrix. This is essential to withstand hydrostatic pressure during F-T cycles [40,41]. After 200 F-T cycles, Group A exhibited substantial cracks and voids, indicating severe internal damage caused by F-T cycles. Conversely, in Group D, fiber pull-out phenomena were observed, accompanied by minor cracks in the matrix. This indicates that HFRC primarily relies on the fracture and pull-out energies of the fibers to dissipate energy [42,43]. As fibers are pulled out from the cement matrix, the energy provided for crack propagation is consumed by frictional stresses, thereby further enhancing the toughness of the HFRC.

Based on the aforementioned analysis, the essence of the damage and deterioration of concrete pavement under freeze–thaw action lies in the evolutionary process of microcrack initiation, propagation, penetration, and ultimately fracture. The incorporation of hybrid SF/BF can enhance the flexural and fracture toughness of concrete, mitigating the rate of crack propagation.

## 4. Grey–Markov Model of F-T Damage

### 4.1. Grey Theory

Drawing upon the theoretical framework presented by Jabeen et al. [44], this section outlines the establishment process of the GM(1,1)-Markov model. This process involves cumulatively generating a sequence model for the RDME loss phenomenon in concrete. Subsequently, this sequence model is subjected to simulation, ultimately yielding predictive values.

Assuming *X*^(0)^(*t*) = {*X*^(0)^(1), *X*^(0)^(2), …, *X*^(0)^(*n*)} represents the irregularly distributed raw data sequence, the application of an Accumulated Generation Operation (AGO) yields *X*^(1)^(*t*) as follows:

(6) $$X^{\left(1\right)}(t)=\left\{\sum_{i=1}^{1}X^{\left(0\right)}(i),\sum_{i=1}^{2}X^{\left(0\right)}(i),\ldots ,\sum_{i=1}^{n}X^{\left(0\right)}(i)\right\}$$

The GM(1,1) model can be expressed by the first-order differential equation as shown in Equation (7).

(7) $$X^{\left(0\right)}(t)+aZ^{\left(1\right)}(t)=b$$

where *a* represents the growth coefficient, *b* denotes the grey input coefficient, and *Z*^(1)^(*t*) pertains to the background value concerning *X*^(1)^(*t*).

*Z*^(1)^(*t*) can be calculated using the following equation:

(8) $$Z^{\left(1\right)}(t)=\frac{1}{2}\left(X^{\left(1\right)}(t)+X^{\left(1\right)}(t+1)\right)$$

Substituting Equation (8) into Equation (7) and using the Laplace inverse transform yields the general solution:

(9) $${\hat{X}}^{\left(1\right)}(t)=\left(X^{\left(0\right)}(1)-\frac{u}{a}\right)e^{-a(t-1)}+\frac{u}{a}$$

Ultimately, by using Equation (9) for cumulative reduction and restoration, the GM(1,1) prediction values can be obtained.

(10) $${\hat{X}}^{\left(0\right)}(t)={\hat{X}}^{\left(1\right)}(t)-{\hat{X}}^{\left(1\right)}(t-1)$$

### 4.2. Markov Chain Correction of Grey Model Errors

Due to the influence of various stochastic factors on the durability degradation trend in concrete pavements in F-T environments, experimental data exhibit significant randomness. This randomness, in turn, affects the predictive accuracy of GM(1,1), leading to less than ideal results. Therefore, to enhance predictive precision, a Markov chain with GM(1,1) was amalgamated.

For the original data sequence, fitting values can be obtained using GM(1,1). Since the residual original values *ε* may have negative values, after applying the absolute value transformation, the residual *ε*^(0)^(*t*) was obtained.

(11) $$\varepsilon^{\left(0\right)}(t)=\left|\varepsilon \right|=\left|X^{\left(0\right)}(t)-{\hat{X}}^{\left(0\right)}(t)\right|$$

Furthermore, applying the steps delineated in Equations (6)–(9) to *ε*^(0)^(*t*) yields the residual prediction model and residual prediction values.

Regarding systematic information within the residual original values *ε*, we can establish a Markov transition matrix to define their states: when the residual original value *ε* is positive, it corresponds to state 1, and when it is negative, it corresponds to state 2. Based on the polarity of the states, we can determine the state transition probabilities.

(12) $$P_{ij}=\frac{M_{ij}}{M_{i}},\;i=1,2;j=1,2$$

The state transition probabilities lead to the derivation of the state probability transition matrix.

(13) $$P=\left[\begin{array}{cc} P_{11} & P_{12}\\ P_{21} & P_{22} \end{array}\right]$$

The remaining computational steps, following the guidelines outlined in Ref. [45], ultimately yield the GM(1,1)-Markov model and its predictive values.

(14) $${\hat{Y}}^{\left(1\right)}(t)={\hat{X}}^{\left(1\right)}(t)+1_{\left\{P(+)\geq P(-)\right\}}{\hat{\varepsilon }}^{\left(1\right)}(t)-1_{\left\{P(+)<P(-)\right\}}{\hat{\varepsilon }}^{\left(1\right)}(t)$$

(15) $${\hat{Y}}^{\left(0\right)}(t)={\hat{Y}}^{\left(1\right)}(t)-{\hat{Y}}^{\left(1\right)}(t-1)$$

**Figure 15:**
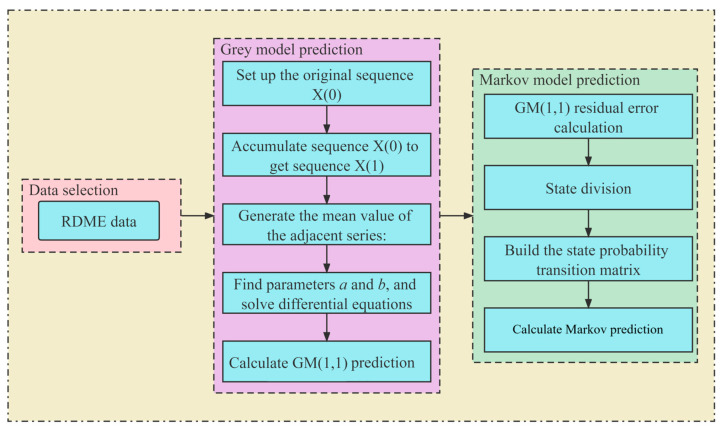
An illustration of the Grey–Markov model based on RDME data.

The computational flow of the Grey–Markov model described above is summarized in Figure 15.

### 4.3. Analysis of Prediction Results

**Figure 16:**
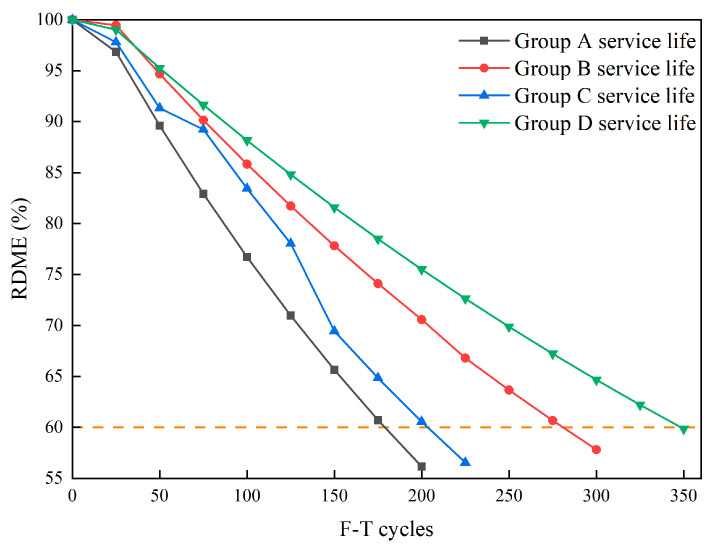
The life prediction curve of FRC specimens based on the Grey–Markov model.

According to the results in Section 3.1.6, the long-term life prediction of the specimen as a function of F-T cycles can be ascertained using the Grey–Markov model, as exemplified in Table 5. It is noteworthy that the correlation coefficients (*R*^2^) lie within the range of 0.9791 to 0.9942, thereby attesting to a commendable concurrence between the projected values and experimental outcomes. Hence, it can be inferred that the utilization of the Grey–Markov model contributes to the anticipation and assessment of the detrimental ramifications inflicted upon HFRC pavement in F-T environments. The corresponding predictive curve is depicted in Figure 16.

In Figure 16, the inverse relationship between the predicted RDME values of specimens and the progressive F-T cycles can be seen. Furthermore, the degradation exhibited an incremental tendency as F-T cycles increased. The RDME damage curve of Group A indicates more severe F-T damage in comparison with the specimens containing fibers, ultimately leading to durability failure after the 175 cycles, aligning with the experimental findings. The F-T damage levels for Groups B, C, and D exhibit a pronounced escalation over the subsequent 50 cycles. As delineated in Table 5, the service life of fiber-reinforced specimens correlates with the number of F-T cycles, amounting to 280 (Group B), 205 (Group C), and 350 (Group D), respectively. Notably, the service life of Group D significantly surpasses that of the remaining specimens. This observation underscores the substantial potential for the application of HFRC in cold regions.

## 5. Conclusions

This study examined the influence of hybrid SF/BF on the durability performance of concrete pavement under varying F-T cycles. The significant achievements of this paper can be summarized as follows:

(1) F-T cycling exerts detrimental effects on the durability characteristics of concrete. Compressive strength, flexural strength, abrasion resistance, and RDME typically exhibit a declining trend with increasing F-T cycles, while the mass loss and damage layer thickness increase with the augmentation of F-T cycles. A copious assembly of randomly dispersed hybrid SFs/BFs within the concrete matrix engenders a three-dimensional constraining framework, thereby efficaciously enhancing the F-T durability of the concrete.
(2) The SEM analysis reveals that the fibers dissipate the energy required for crack propagation by means of friction with the cementitious matrix, as well as the pull-out energy and fracture energy of the fibers, thereby serving to toughen and impede crack propagation, consequently enhancing F-T resistance. The microhardness test results indicate that the ITZ strength is lowest in the control group, whereas in the HFRC, the impact of F-T cycles on the ITZ is relatively minimal due to the robust bonding between fibers and the surrounding matrix.
(3) A Grey–Markov model, built upon the results obtained from the RDME test, is formulated to predict the service life of each group of specimens. The hybrid method affects the concrete’s service life. Under F-T cycles, the predicted life of each group in the sequence is Group D > Group B > Group C > Group A.

This study conducted initial endeavors in the application of hybrid steel fibers and basalt fibers in concrete pavement. Prior to on-site implementation, further concrete mix experiments, primarily encompassing the design optimization of fiber volume fractions, should be undertaken. Considering aspects such as mechanical performance, crack width control, economic viability, and environmental conditions, the ideal blend involves steel fiber volume fractions below 2% and basalt fiber fractions below 0.2%. Additionally, the potential mechanisms by which the hybrid fibers control cracks warrant further investigation.

## Figures and Tables

**Table 1:** Chemical composition of cementing materials (/%).

Chemical Composition	SiO_2_	Al_2_O_3_	CaO	Fe_2_O_3_	MgO	MnO	K_2_O	IL	TiO_2_
Cement	26.7	11.5	48.9	4.9	3.0	0.4	1.6	1.8	1.2
Fly ash	46.4	29.9	9.4	6.9	1.9	0.2	1.5	2.4	1.4

**Table 2:** Physical and mechanical properties of the fibers used in this experiment.

Types	Length (mm)	Diameter (μm)	Density (g/cm^3^)	Elastic Modulus (GPa)	Tensile Modulus (GPa)
SF	30	50	7.8	200	1.2
BF	12	20	2.7	100	4.5

**Table 3:** Mix proportions of concrete specimens.

Samples	A	B	C	D
Cement/(kg·m^−3^)	400	400	400	400
Fly ash/(kg·m^−3^)	100	100	100	100
Coarse aggregate/(kg·m^−3^)	1165	1165	1165	1165
Fine aggregate/(kg·m^−3^)	635	635	635	635
Water /(kg·m^−3^)	200	200	200	200
Water reducer/(kg·m^−3^)	4.5	4.5	4.5	4.5
SF/% (by volume fraction)	/	2.0	/	2.0
BF/%(by volume fraction)	/	/	0.1	0.1

**Table 4:** Dimension of concrete specimens.

Test Project	Specimen Dimension/mm
Mass loss	100 × 100 × 100
Abrasion resistance	150 × 150 × 150
Compressive strength test	100 × 100 × 100
Flexural strength test	400 × 100 × 100
Damaged layer thickness	400 × 100 × 100
Relative dynamic modulus of elasticity	400 × 100 × 100

**Table 5:** The prediction of service life for FRC specimens.

Samples	*a*	*b*	*R* ^2^	Expected Service Life (Time)
A	0.077	109.810	0.9791	175
B	0.048	105.972	0.9932	280
C	0.068	110.108	0.9889	205
D	0.039	105.395	0.9942	350

## Data Availability

Data are contained within the article.
